# Study of methods for endpoint aware inspection in a next generation firewall

**DOI:** 10.1186/s42400-022-00127-8

**Published:** 2022-09-03

**Authors:** Jenny Heino, Antti Hakkala, Seppo Virtanen

**Affiliations:** 1 Department of Computing, University of Turku, Turku, Finland; 2 Forcepoint LLC, Helsinki, Finland

**Keywords:** Network traffic, Endpoint identification, NGFW, Endpoint aware inspection

## Abstract

Given the global increase in remote work with the COVID-19 pandemic and deperimeterization due to cloud deployment of next generation firewalls, the concept of a next generation firewall is at a breaking point. It is becoming more difficult to define the barrier between the good and the bad. To provide the best security for an endpoint with minimal false positives or false negatives it is often necessary to identify the communicating endpoint application. In this study, we present an analysis of key research and methods for providing endpoint aware protection in the context of a next generation firewall. We examine both academic research as well as state-of-the-art of the existing next generation firewall implementations. We divide endpoint application identification into passive and active methods. For passive endpoint application identification, we study several traffic fingerprinting methods for different protocols. For active methods we consider active scanning, endpoint metadata analysis and content injection and reference existing implementations. We conclude that there are several open areas for future research, and that none of the considered methods is a silver bullet solution for endpoint aware inspection in the context of a next generation firewall. To our best knowledge, this is the first study to examine current research and existing implementations of endpoint aware inspection.

## Introduction

A traditional firewall is a network technology solution installed at the boundary of two networks. It controls which connections are permitted through and which are not, based on network and transport layer protocol information. These solutions are sometimes also referred to as Packet Filter Firewalls or first generation firewalls (Cisco Documentation 2020). Traditional firewalls have existed since around 1987 (Ingham and Forrest 2002). In the early days of the internet, these solutions provided sufficient protection against intruders by blocking unwanted access based on IP addresses and port numbers. However, as the network landscape became more and more complex with the implementation of sophisticated web applications, the shortcomings of these traditional firewalls became obvious. Eventually, these shortcomings, especially in the lack of visibility into the application layer data, brought about the rise of more advanced solutions.

The term *Next Generation Firewall* or *NGFW* was first used by Palo Alto Networks in 2010 (Brazil 2020; Gold 2011). A next generation firewall refers to a network technology solution which enhances traditional firewall technologies with additional features that especially focus on *deep packet inspection*. Deep packet inspection refers to the ability of the network technology solution to process the “deeper” layers of the traffic which are transported on top of the transport layer protocols. With the support of deep packet inspection, an NGFW is able to provide advanced security capabilities. These include *Intrusion Prevention Systems* or *IPSs*, controlling the network traffic based on *network applications*, and increased visibility and security by being able to decrypt TLS traffic. In this context, the term “network application” refers to different web service providers (such as Google or Facebook), but it also encompasses protocol identification on the application layer. NGFWs are often also able to implement security policies based on user and group IDs. In addition to the ability to control traffic based on transport and application layer properties, Next Generation Firewalls provide lower level features, such as routing and NATting of traffic. Many NGFWs also provide IPsec VPN or SSL VPN capabilities (Neupane et al. 2018; Thomas Skybakmoen 2022).

NGFWs have traditionally been developed and deployed as separate physical appliances of varying sizes, depending on the performance requirements of the end user. The amount of desired features affects the traffic throughput of an NGFW appliance. As an example, features such as threat prevention and VPN can affect the throughput of the NGFW, resulting in the need of a larger appliance than would be required for simple Firewall functionality (Palo Alto Networks Inc 2022a; Fortinet Inc 2022a; Cisco and/or Its Affiliates 2022; Forcepoint 2022).

Similarly to traditional firewalls, Next Generation Firewalls have typically been located at the barrier of the “good” office network and the “bad” internet. However, this convention is at a breaking point as it is becoming increasingly common for the employees to access the office network using remote workstations or mobile devices. Most NGFW vendors are developing capabilities for NGFW instances in the cloud, or *Firewalls as a Service (FWaaS)*. More dimensions are also added with *Software-defined Networking in a Wide Area Network (SD-WAN)* capabilities. The latest development is the introduction of *Secure Access Service Edge* or *SASE* by the research and advisory company Gartner in 2019 (Lerner 2020). SASE combines products like FWaaS and SD-WAN to acknowledge the need for a remote security solution for the constantly growing remote workforce. As the field becomes more complex, the barrier between the good and the bad is becoming more difficult to define.

The term *endpoint* refers to a computer or another entity which communicates with other entities over a network. These endpoints are usually personal computers, server machines, mobile devices or IoT devices. In the context of this study, *endpoint applications* refer to the software components on an endpoint that communicate with other software components over a network. These include client applications such as web browsers and email clients, but also server applications such as web and email servers.

When inspecting network traffic, there are patterns of traffic that can easily be considered “good” or “bad”, no matter what endpoint application is at the receiving end. However, there is a gray area in between, where false positive or false negative identifications become an issue. The same network traffic may be benign to one endpoint application but be processed by another in a way that may compromise the entire endpoint. On one hand, without knowledge on the communicating endpoint applications, the risk of false positives or false negatives is heightened. On the other hand, traffic that triggers a vulnerability in one endpoint application may be extremely common or even crucial for another. Terminating this kind of traffic for all endpoint applications may thus prevent a particular application from working properly. Because of this, some knowledge on the endpoint applications can be crucial for separating malicious traffic from normal.

In the conventional NGFW context where an NGFW inspects the traffic at the barrier of the office network and the internet, this can be controlled to some extent by an administrator using traditional firewall methods such as restricting or enforcing the inspection functionalities based on the static IP address of an endpoint. This method is, however, not exhaustive as different endpoint applications, such as different browsers, may process the same type of traffic on one endpoint. A “good” endpoint may also become “bad”—it may for example get compromised due to a malware infection, in which case a static white-listing of the endpoint may cause a larger breach.

*Endpoint agnostic inspection* refers to an inspection process where no additional information about the endpoint application is used when making the decision of permitting or terminating traffic. The advantage of applying endpoint agnostic inspection is in identifying clearly malicious traffic. When an actual attack is identified by an NGFW, it does not matter if the target was vulnerable to the attack or not—the NGFW should identify and block the attack nonetheless. An administrator needs to be aware of an active exploit in the network even when there are no vulnerable endpoints. *Endpoint aware inspection* refers to an inspection process where information about the endpoint application is used when making the decision whether the traffic should be permitted through or not.

Endpoint aware inspection becomes necessary when considering the previously mentioned grey area of network traffic, and the false positive and false negative identifications that happen in that area. When a traffic pattern is identified to indicate an attack against a certain vulnerable endpoint application, but the same traffic pattern is also present in benign traffic when it is not directed to the vulnerable endpoint application, an NGFW without endpoint awareness is faced with a difficult decision. On one hand, the NGFW can block that traffic pattern from all traffic, to protect the vulnerable endpoint application, but potentially affecting benign traffic for other endpoint applications on the way. On the other hand, the NGFW can permit that traffic pattern for all traffic to ensure functionality for other endpoint applications but leaving the vulnerable endpoint application at risk. When the NGFW is able to perform endpoint aware inspection, it does not need to make that decision—it can block the traffic when it is directed at the vulnerable endpoint application, and permit it for all other endpoint applications. This gives the NGFW a greatly enhanced ability to reduce the potential false positive and false negative identifications that arise from the network traffic.

The Zero trust paradigm (Kindervag 2010) introduced in 2010 aims to improve network security by breaking previous assumptions on the trustworthiness of the protected internal network. While traditionally firewalls have been used to partition networks into trusted and untrusted networks, zero trust assumes that external and internal threats are present on the network all the time, and that a network does not become trusted by the virtue of being an internal network. The implementation of zero trust networks requires advanced capabilities from firewalls and intrusion detection systems. NGFW solutions are needed to provide information on all network resources in order to facilitate the realization of the zero trust paradigm in monitored networks. Endpoint aware inspection is another tool for NGFWs with which to analyze and monitor network resources in both internal and external networks.

**Fig. 1:**
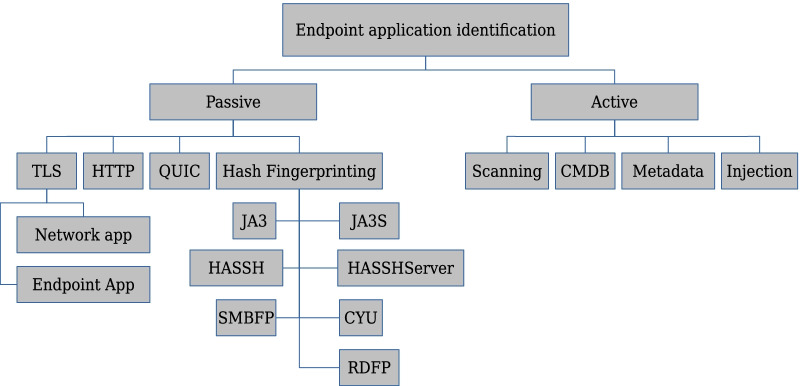
Endpoint application identification taxonomy as covered in this paper

In this study, we present an analysis of the key research on methods of providing endpoint aware protection in the context of a Next Generation Firewall. We divide endpoint application identification methods into two categories: passive and active. Figure 1 presents a taxonomy for endpoint application identification as covered in this paper. These methods were selected based on their applicability in the context of a Next Generation Firewall. The selected passive identification methods are based on deep packet inspection and other methods viable for an NGFW, and the active methods mostly rely on external components with existing NGFW integrations as well as methods already implemented in several NGFW products. Of the methods considered in this paper, content injection is the only one that is currently only a concept. No known implementations exist for it.

We examine both academic research as well as state-of-the-art of existing NGFW implementations. Based on this analysis, we identify the open areas in this field where future research should focus. To our best knowledge, this is the first study to examine current research and existing implementations of endpoint aware inspection.

## Passive identification

On one hand, identifying an endpoint application passively from network traffic requires no interaction with the endpoints. If accurate passive identification is possible, it could be the most beneficial method in the context of an NGFW. With passive identification, the protection scope is also the greatest: it encompasses all endpoint types including mobile devices as well as desktop and server machines.

On the other hand, passive identification exposes a security issue: if the NGFW can passively identify an endpoint application from network traffic, so can anyone else with visibility to the traffic. With the information that the NGFW uses to protect vulnerable endpoints, a malicious entity can likewise hunt for vulnerable targets. The security foundation of an NGFW should not contradict with the overall security of the endpoint.

To be able to perform passive identification as described in this section, an NGFW needs to be able to perform deep packet inspection on the addressed network protocols. Different network protocols have different issues regarding false positive and false negative identifications. As an example, many network protocols have the ability to transport files, and files are one source for potential false positive and false negative identifications. However, different NGFW vendors have different levels of support for extracting and performing analysis on files from different network protocols. Also, this kind of vendor-specific information is not fully openly disclosed by vendors. Therefore, in this paper, we do not take a deeper look into what protocols, and in what depth, different NGFW vendors are able to perform deep packet inspection on. This level of detail is out of the scope of this study.

In this section, we consider the current research on passive endpoint application identification from the network traffic and the existing methods. In section “HTTP”, we briefly consider identifying the endpoint application based on the User-Agent string in the HTTP header. In section “TLS”, we take a deeper look into the current research on identifying the endpoint application from TLS traffic. In section “QUIC”, we take a look at the QUIC protocol. In section “Other protocols”, we consider the current research regarding other protocols. In section “Hash fingerprinting”, we consider the recent development in the field of endpoint application identification from network protocols using hash fingerprints. Finally, in section “Mobile application”, we survey the current research on mobile application identification.

### HTTP

Since the context of this study is to find ways for providing endpoint-aware security for vulnerable endpoint applications, using the User-Agent string in the HTTP header for identifying the browser application (Mozilla Foundation 2020a; Microsoft Corporation 2020a; Google 2020a) can be considered a sufficient method for identifying the endpoint application from plain HTTP. Although various browsers allow spoofing the User-Agent field (Microsoft Corporation 2020b; Google 2020b), this can be considered an intentional attempt to evade security, which is not in the scope of this study.

The most popular browser, Google Chrome (Wikimedia Foundation 2020; Net Applications 2020; StatCounter 2020), has expressed plans to freeze the User-Agent string (Google 2020c). This means removing platform and version specific information from the User-Agent but leaving the browser specific information. It remains to be seen how this will affect the content of the User-Agent field on other platforms.

### TLS

TLS is the most relevant subject for research due to its popularity. The latest reports from Google (2020d) show that, on average, over 80% of the pages loaded with the Chrome browser use HTTPS. Because of their wide usage, web browsers are also a major target for attackers. The number of published security vulnerabilities in 2019 for the Google Chrome browser was 177 (Google 2020e) and for Mozilla Firefox 105 (Mozilla Foundation 2020b).

Identifying the endpoint application from encrypted TLS traffic can be useful in the context of an NGFW, especially when considering routing or basic access control. When considering the issue of providing the best security for a vulnerable endpoint application, in most cases it can be assumed that the traffic can be decrypted by the NGFW and the tunneled traffic can be inspected as is. Some exploits, however, such as FREAK (miTLS Team 2020), Logjam (Adrian et al. 2015) and POODLE (The OpenSSL Project 2020), aim at vulnerabilities in the TLS implementation on the endpoint application. In this section, we will review the current research on identifying the application from encrypted TLS.

#### Network application

Identifying the network application from encrypted TLS traffic, especially in the case of HTTPS, has been extensively studied. A vast collection of different machine learning and deep learning techniques have been proven to be extremely effective in classifying TLS traffic (McCarthy and Zincir-Heywood 2011; Rezaei and Liu 2019; Shbair et al. 2016). Although identifying the Network Application is not in the scope of this study, it should be noted that when considering future research, similar methods may be useful for identifying endpoint applications.

#### Endpoint application

Identifying the endpoint application from TLS traffic has not been as extensively studied as identifying the network application. Identification of the endpoint client application, most often the web browser, has been explored in some papers. A common element in almost all papers on TLS identification is the use of supported cipher suites, either alone or together with other identifiers, to identify the underlying endpoint application.

In an early study on SSL identification from 2007, Bernaille and Teixeira investigate identifying the application layer protocol inside an SSL tunnel based on the first few packets (Bernaille and Teixeira 2007). The method relies on the size of the first three encrypted application packets, taking into account that different encryption algorithms result in different packet sizes. The researchers used encrypted traffic of HTTP, POP, FTP, Bittorent, and Edonkey. The accuracy reached over 90% for all other protocols but Bittorrent, which reached 78% accuracy.

The research in Bernaille and Teixeira (2007) only focuses on SSL, so its applicability for current network traffic using TLS is unclear and requires further research. Despite the research being partially outdated, it introduces a simple but interesting method for separating encrypted web traffic from encrypted non-web traffic. This can help an NGFW to separate HTTPS traffic generated by a web browser from other traffic that is tunneled over TLS, thus better focusing the inspection features correctly.

Husák et al. (2016) explore the method of mapping the list of supported cipher suites provided in the Client Hello message to the User-Agent in the HTTP message. To achieve this, the authors used two methods: first, they created a test TLS server for harvesting accurate results by collecting the User-Agent information from decrypted TLS connections, and second, they observed student network traffic and correlated HTTP and HTTPS connections initiated from the same endpoint roughly at the same time.

Husák et al. first observed that a small amount of unique lists cover a great part of all TLS traffic: the top 31 of the observed 1598 unique lists cover over 90% of all traffic. They also found that several User-Agent strings often map to one list of cipher suites. For each list, the authors selected the most commonly matching endpoint application and endpoint OS. Using this method the authors were able to identify at least one of the two with a 95.4% accuracy. For only 4.6% of the observed TLS traffic, neither the endpoint OS nor the endpoint application could be identified. However, the endpoint application alone could not be identified for about 16% of the traffic, yielding about 84% accuracy for endpoint application identification with this method.

The disadvantage of the research by Husák et al. (2016) is the inaccuracy of the second method for mapping the cipher suite lists with the HTTP User-Agents: several client applications usually initiate HTTP and HTTPS connections from one client machine, which can lead to false correlations. Nevertheless, this method is very simple, and the results obtained in their study show good promise for this method to be easily applicable in the context of an NGFW. This method, however, provides no visibility to the server application.

A similar, but more detailed, method for endpoint client application identification from network traffic alone, is presented by Muehlstein et al. (2017). They explore the passive identification of the operating system, browser and network application from encrypted HTTP traffic. They derive a detailed feature set from a TLS connection. It includes information on the TLS connection such as the supported cipher suites, compression methods and extension count, but also information on the traffic flow such as amount of packets and bytes and information on traffic bursts. This feature set accompanied by machine learning [*Support Vector Machine* (SVM) (Cortes and Vapnik 1995) with *Radial Basis Function* (RBF)] provides an identification accuracy of 96.06%. For generating the dataset, the authors used Selenium Web Automation tool (The Selenium Project 2019).

The research focuses on TLS traffic in port TCP/443 and on the identification of browsers, with references to “Non-Browser” traffic for so-called Microsoft-Background traffic. Nearly 10% of the traffic in their test is produced by these non-browser endpoint applications. Due to the selected feature set, this method is applicable only after the whole connection has been processed. In the context of an NGFW, post processing may be useful for traffic analyses such as network traffic reports, but it is not a feasible solution for inline security. Again, the research does not cover identifying the server application.

### QUIC

The QUIC protocol is an encrypted multiplexed stream transport protocol over UDP, originally designed by Google (Roskind 2020). The usage of QUIC protocol is constantly growing in the internet. In Rüth et al. (2018), the researchers show that QUIC accounted for 2.6–9.1% of the traffic in the internet in 2017, with Google using QUIC for 42.1% of its traffic, and Sy et al. (2019) mention that approximately 7% of global internet traffic in 2018 was QUIC. In Sy et al. (2019) it is also observed that 186 of the Alexa Top Million sites (Alexa Internet Inc 2020) had QUIC support in 2018.

As the QUIC protocol was adopted by IETF and standardized in RFC 9000 (Iyengar and Thomson 2021), the term *gQUIC* became a common term for referencing the original protocol specification from Google. The IETF standardized version of QUIC utilizes TLS 1.3 inside the QUIC packets, and the use of HTTP inside the IETF standardized QUIC has been titled HTTP/3 (Bishop 2019).

**Fig. 2:**
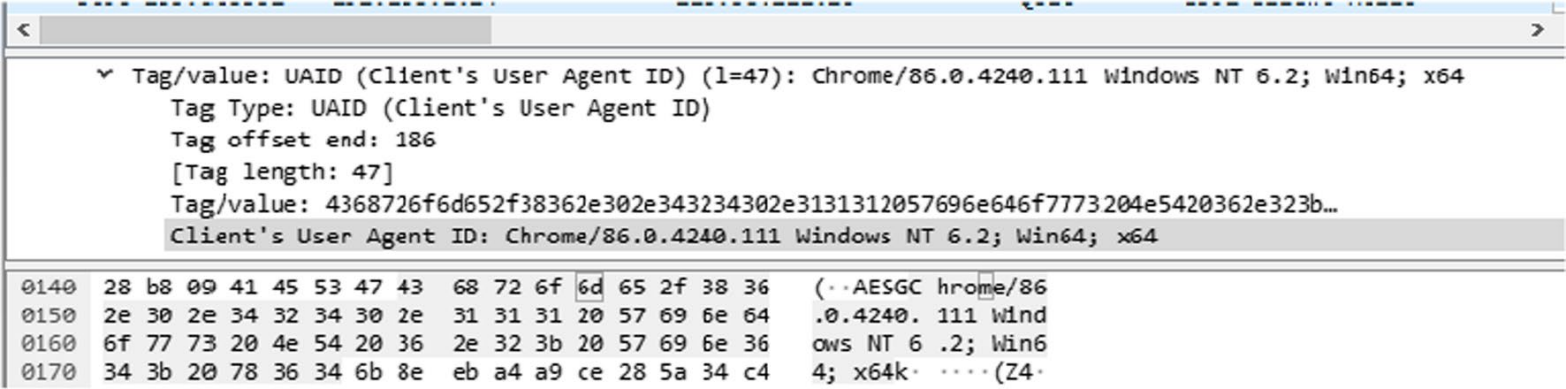
gQUIC user agent value in the unencrypted Client Hello message from the latest version of Chrome browser at the time of writing (86)

Despite being an encrypted protocol, the gQUIC protocol introduces the client’s User Agent ID value in the unencrypted Client Hello. Shah (2018) finds that it is possible to identify the endpoint applications and operating systems from the network based on the gQUIC User Agent value. It is noteworthy that this field is an optional field, and most implementations do not include it in the Client Hello (Lastovicka et al. 2018). However, in the latest version of Chrome browser at the time of writing (86), the User Agent value is included in the unencrypted Client Hello, as seen in Fig. 2.

Due to being a relatively new addition to the common web protocols, not much academic research has yet been released on identifying endpoint information from QUIC traffic, when not taking the User Agent into account. This is a severe lack in the current research, due to the rapidly growing popularity of the protocol. In addition, as HTTP/3 will use QUIC, the importance of further research on QUIC increases.

### Other protocols

HTTP and TLS comprise a large part of all internet traffic, which explains their great coverage in current research. An NGFW is, however, often used for inspecting many other protocols as well, and protecting vulnerable endpoint applications that process network traffic using these protocols. Identifying the endpoint applications from other protocols than HTTP and TLS is also a major area of interest.

Many protocols contain short simple byte patterns, or magic bytes, that provide a sufficient way of identifying the protocol itself. In addition, there are many papers providing other methods for identifying the application layer protocol. In Yun et al. (2016), the researchers present a tool called Securitas, which they show to be able to identify the protocol to a 98.4% accuracy. It does not, however, identify the endpoint application producing the traffic.

Identifying the endpoint application from other protocols than HTTP or TLS has not raised much academic interest. Despite being unencrypted, there is no simple method, like the User-Agent is for HTTP, for mapping the endpoint application for many common protocols such as DNS, FTP, and SMB. The need for identifying the underlying endpoint application, however, still exists in the concept of an NGFW. The vulnerabilities in the client and server implementations for certain protocols, such as SMB or DNS, may lead to a massive system compromise. A good example is the WannaCry ransomware campaign in 2017 (Mohurle and Patil 2017), which propagated exploiting a vulnerability (Microsoft Corporation 2020c) in the older implementations of the SMB protocol on Windows systems. Despite there not being much academic research on the endpoint application identification for other protocols, several methods for fingerprinting the endpoint application from multiple different protocols have recently been developed. These methods are explored in the next section.

### Hash fingerprinting

Endpoint application fingerprinting, by calculating a hash value from certain protocol details, is a fairly recent, but active field of study. An established method for creating a fingerprint is to generate an MD5 hash from a suitable set of significant and distinctive values from the protocol fields. Several methods for different protocols have been proposed, and more methods are constantly being developed, but little academic research has been published on their effectiveness. However, case studies have shown that the proposed methods show enough promise to justify the need for further research. In this section, we present the current status of endpoint application fingerprinting utilizing this method.

#### JA3 and JA3S fingerprinting

The first endpoint application fingerprinting methods utilizing the hash fingerprinting method were the JA3 and JA3S fingerprints. JA3 and JA3S are methods for fingerprinting TLS handshakes, developed by Salesforce employees John Althouse, Jeff Atkinson and Josh Atkins, and open-sourced by the company in 2017 (Althouse et al. 2020a). The method is based on TLS fingerprinting research by Lee Brotherston, presented in 2015 at DerbyCon (Brotherston 2015).

In essence, the JA3 fingerprint uses similar methods as the studies discussed in subsection TLS. To generate a JA3 fingerprint from the Client Hello message of a TLS handshake, the following values are stored in a specific format: Version, Accepted Ciphers, List of Extensions, Elliptic Curves, and Elliptic Curve Formats. An MD5 hash value is then taken of the stored value to obtain the JA3 fingerprint. Similarly, the JA3S fingerprint is generated from the Server Hello message by storing the values for Version, Accepted Cipher, and List of Extensions, and taking an MD5 hash value of the result.

Despite being a somewhat recent invention, there has already been some academic research published on the JA3 and JA3S fingerprints. In Truong (2019), Ai Truong evaluates the accuracy of JA3 and JA3S for identifying TLS connections. The research focuses on identifying malicious traffic from normal traffic, but it also has some valid points on JA3 accuracy when considering the issue in general. The research shows that, to separate malware connections from legitimate traffic, more accurate results are received from combining the use of JA3 and JA3S fingerprints than from using JA3 alone. It also shows that using machine learning techniques, more specifically decision trees, on the values that are stored for the JA3 and JA3S fingerprints, provides more accurate results than the fingerprints themselves. The research concludes that a big issue with JA3/JA3S fingerprinting is that many applications use the same underlying TLS libraries for generating the TLS connections, which is the main reason for false identifications.

The main weakness of the JA3 and JA3S fingerprints, which causes the large amount of collisions, is that they leave out most of the information that could be gained from the extensions in the TLS handshake messages. Some extensions, such as the Server Name Indication extension in the Client Hello message, are clearly web service specific, and using their value when generating the fingerprints would only reduce the usability of the fingerprints. Many of the extensions are, however, not web service specific in the same way, and could be used to make the fingerprints more precise.

The JA3 fingerprinting method is, however, a lot more thorough than the method of only taking the list of the supported cipher suites used in Husák et al. (2016). This leads to the assumption that JA3 fingerprints should lead to more accurate results than what was achieved in Husák et al. (2016). The JA3S fingerprint is also the only method for identifying the server application that was encountered during this study. It is clear that more research on the accuracy and applicability of the JA3 and JA3S fingerprints is needed.

#### Hash fingerprinting for other protocols

After the release of the JA3 and JA3S fingerprinting methods, many similar fingerprinting methods have been introduced for different protocols. Many of them show promising preliminary results. Due to the cutting-edge nature of these methods, however, no academic research has yet been published on their effectiveness.

Methods for fingerprinting SSH client and server applications, called HASSH and HASSHServer, were developed by Salesforce employee Ben Reardon and open sourced by the company in 2018 (Reardon 2020). The HASSH and HASSHServer fingerprint is calculated from the clear-text *SSH_MSG_KEXINIT* messages sent by both the client and the server. The fingerprint utilizes the set of supported and preferred algorithms listed in these messages. These values are first stored in a specific format, and an MD5 hash value is then taken of the stored value. The author presents preliminary results which indicate that the method can be especially useful for identifying malicious connections, but they also show that it can be used for identifying legitimate connections.

A method for fingerprinting gQUIC clients, called CYU, was developed by Salesforce employee Caleb Yu and open sourced by the company in 2019 (Yu 2020). To generate the CYU fingerprint, the version, and a list of the tag values, are collected from the gQUIC Client Hello message. An MD5 hash is then calculated from the collected values, which constitutes the CYU fingerprint. A simple use case presented in the publication indicates that the CYU fingerprints can be used for identifying malicious actors, but further research is needed to verify whether the method can be used for providing endpoint aware inspection in the context of an NGFW.

RDP fingerprinting is explored by Karimishiraz (2020) published in 2019. The author notes that RDP clients that use the Enhanced Security mode can be fingerprinted using the JA3 fingerprinting method. For fingerprinting RDP clients that use the Standard Security mode, they introduce a preliminary method called RDFP. The method is described as experimental, and susceptible for modifications before final release. The method utilizes certain values collected from the clientSecurityData, clientClusterData, clientNetworkData and clientCoreData structures sent during the Basic Settings Exchange phase of the connection, and calculates an MD5 hash of these values. Due to being in the early stages of development, no further publications on RDFP were found during this study. A similarly preliminary method for fingerprinting SMB, called SMBFP, is introduced by Torres (2020) published in 2020. This method is described as being *incredibly bleeding edge* by the author, and no preliminary results on the method’s effectiveness have yet been published.

### Mobile application

Identifying mobile applications based on the network traffic is explored in several papers. Although identifying mobile applications is useful for an NGFW, the main focus is on local workstations and laptops. In this section we briefly touch on the current research on mobile application identification.

In Shbair et al. (2016), the researchers introduce NetworkProfiler, a tool for identifying a mobile application based on HTTP traffic. The tool utilizes *UI fuzzing* for profiling the application traffic. UI fuzzing is the method of automatically generating different UI events that trigger new behavior, and thus new network traffic patterns, from the application. NetworkProfiler suffers from being limited to unencrypted traffic. In Taylor et al. (2016) the researchers present AppScanner, which also uses UI fuzzing for traffic generation. AppScanner focuses on TCP traffic, but it does not look into the packet payloads. This makes the tool application-layer protocol agnostic: AppScanner is not restricted to HTTP or HTTPS traffic. It analyzes traffic bursts, meaning all network packets from the device within a certain time frame, and traffic flows, meaning sequences of packets within a burst with the same destination IP address and port number. Based on these, AppScanner generates application fingerprints. The paper explores several different classification methods, and provides performance numbers for the different methods. Despite being presented as a mobile application identifier, Taylor et al. point out that due to its modular design, it should be easily portable to other platforms as well.

In Taylor et al. (2018) the researchers further expand the research on AppScanner, by analyzing its accuracy with different application versions and time passage. They also present a method for separating ambiguous traffic, or traffic that is common among different applications, from the dataset. The research shows that, if no post-processing or removal of ambiguous traffic is done, the performance of the method is not too impressive. The best result was achieved when the device and application version remained the same over the course of six months, yielding a 40.9% detection accuracy. The accuracy decreased when the device or the application version changed. After applying post-processing and removal of ambiguous traffic, the method reached 73% accuracy in the worst-case test and 96% accuracy in the best case test.

Due to the research being restricted to a mobile device, it is difficult to estimate how the methods used by AppScanner would work for an NGFW. In the context of an NGFW, the method of fingerprinting the traffic bursts and traffic flows in this way, however, again restricts the method only to post processing tasks such as traffic reports. For inline processing, the identification needs to happen earlier in the process. The UI fuzzing methods introduced in Shbair et al. (2016) and Taylor et al. (2016), however, provide an interesting ground for further research on endpoint application identification on other platforms.

## Active identification

As opposed to passive endpoint application identification from the inspected traffic, endpoint application information can also be received from an external component. With active identification, some amount of interaction with the endpoint is always required. Passive methods have to rely on the assumption that the endpoint is not trying to evade identification—it is always possible for an endpoint application to fake traffic patterns that make it look like another endpoint application. In this sense, active identification methods are more resilient. However, it is not always possible to enforce active methods, if they require external software components being installed on the endpoint. Some active methods may also provide out of date information.

In this section, we consider the current research and state-of-the-art implementations on active endpoint application identification methods. In section “Active scanning”, we introduce active scanning, which can be used for mapping the server applications on an endpoint. In section “Configuration management database”, we take a look at configuration management databases, and integrating them into an NGFW. In section “Endpoint Metadata”, we consider receiving endpoint application information in the form of connection metadata from an external component installed on an endpoint. Finally, in section “Content Injection”, we briefly explore the concept of modifying the traffic payload in a way which prevents an endpoint application from executing potentially malicious content before the NGFW has approved it.

### Active scanning

Most passive identification methods focus on identifying the client application. To identify a client application, the client first needs to initiate a connection. This cannot, in most cases, be forced by an external component, such as the NGFW. However, with server applications, the NGFW can actively initiate connections to obtain information on the server processes.

The methods for identifying an endpoint application based on the network traffic are the same with passive and active identification. For example, JA3S fingerprints can also be used for identifying the TLS server with active scanning methods. However, active scanning requires more functionality on the NGFW, since in many cases it needs to form a valid client message for the server to reply with a valid message. In addition, with some protocols such as TLS, the server reply depends on the initial client message (Rescorla and Dierks 2008), which can complicate version identification.

To perform a comprehensive active scan on a network may also be time consuming, depending on the scope of the scan. The information can also become obsolete, if the scans are not performed frequently enough. In the best scenario, information received using active scanning can be complemented with information received using passive scanning, thus making the need for a new active scan less frequent.

The most vast open source implementation of an active server version scanner is Nmap (Lyon 2020a). In addition to basic port scanning using TCP SYN and TCP ACK packets, it can also perform OS and version identification. Nmap can currently identify over 2200 well-known services (Lyon 2020b). At least the Cisco Firepower NGFW can utilize Nmap output in its configuration (Cisco Systems Inc 2020a).

The best known commercial product for active scanning is the Nessus vulnerability scanner (Tenable Inc 2020a). Nessus can perform different types of scans ranging from basic port scanning to specific vulnerability scanning. Many NGFW vendors have integrated support for Nessus reports. NGFW implementations with Nessus integration include Fortinet NGFW (Tenable Inc 2020b) and Cisco Firepower (Cisco Systems Inc 2020b).

A recent, but noteworthy, addition to the active scanners is the JARM fingerprinting tool for TLS servers, published and open sourced in 2019 by Salesforce employees John Althouse, Andrew Smart, RJ Nunnally and Mike Brady (Althouse et al. 2020b). When creating a fingerprint of the server, JARM sends 10 specifically crafted client requests to the server with the intention of finding out how the server responds to different configurations. The authors note that JARM can, as an example, be used for identifying when a target server is not running the intended latest TLS configuration.

### Configuration management database

A *Configuration Management Database*, or *CMDB*, is a database, which contains information on the organization’s information systems. These assets include hardware and software assets, but may also reference other assets, such as people, or documentation. The assets are commonly referenced as Configuration Items (CI) (Rouse 2020).

Existing CMDB implementations include passive and active systems. In a passive CMDB system, an administrator will manually import and update information for the assets. An active CMDB will have the ability to scan the network for existing assets. Most passive CMDB systems have the ability to integrate active scanners to reduce the required manual labor. However, all CMDB systems will require some amount of manual work, as information such as installation date and client application information is rarely available with active scanning.

Integrating a CMDB into an NGFW can provide a static source of information for the endpoint applications installed on an endpoint. The challenge in this approach is keeping the CMDB up to date in relation to the installed endpoint applications. The efficiency of this approach depends heavily on how strictly the organization controls which endpoint applications the users can install on their endpoints, and how often and through which method the CMDB is updated.

### Endpoint metadata

The most accurate way to identify the endpoint application associated with a network connection on an NGFW is having the endpoint provide this information to the NGFW. This can be achieved by installing an external software component on the endpoint, which provides metadata over a secure connection of each new network connection to the NGFW. In this scenario, no deep inspection is needed to identify the endpoint application. In addition, this method does not require that the endpoint compromises its security also for other observers beyond the NGFW.

This method is restricted to endpoints that can be controlled by the administrator, so that external software components can be installed on the machine. In many scenarios, this can not be expected. The endpoints that can be monitored in this way, do, however, provide a unique platform for collecting information on the network traffic produced by different endpoint applications. Since the source of the connection is reliably known, certain information on the network traffic patterns, such as hash fingerprints, can be accurately mapped to the endpoint application. This information can then be further used for identifying traffic on endpoints that do not have the external component installed.

Automatically collecting traffic pattern information from these environments provides a robust way to get up-to-date mappings between traffic patterns and endpoint applications. On one hand, an NGFW can control endpoints that provide metadata, and endpoints that do not. The NGFW could then locally leverage the information collected from the endpoints that provide the metadata to identify the endpoint application from the traffic on the endpoints that do not. On the other hand, if this information can be provided as telemetry data to the NGFW provider, the information can be dynamically distributed to other NGFWs as well. When providing this information as telemetry, it needs to be taken into consideration that no unnecessary or personally identifiable information is included.

### Content injection

One angle to endpoint aware inspection is to outsource the content parsing to the endpoint application itself, and let it report to the NGFW on what is about to be executed. This ensures that the decision to block or allow traffic is made with the exact content that the endpoint application will process. This can be achieved, for example, by the concept of content injection. With content injection, the traffic is modified in a way which makes the endpoint application process the traffic slightly differently than what would have happened with the original content. The aim is to make the endpoint application process the content in its entirety only after the NGFW has given its verdict.

One method for doing content injection is Distributed Client Protection (Jalio et al. 2020). In this method, a security device, such as an NGFW, injects additional content to the original content which overrides certain functionalities in it. When the endpoint receives the content, instead of executing it, the overridden executive function reports what is about to be executed to the NGFW. The NGFW can then evaluate what is about to be executed, and communicate to the endpoint if it can execute it or not.

This injection method is especially powerful against certain file obfuscation methods. Many targeted attacks use scripting methods that are executed in a different way by a vulnerable application, hiding this behavior under several different layers of obfuscation. Using the content injection method, the NGFW sees exactly what is going to be executed by the client, providing targeted protection only to the endpoints that would get compromised by the content.

Another benefit is that, with this method, no active configuration is needed on the endpoint. This means that an NGFW can automatically provide protection to all endpoint applications that process the traffic where additional content is being injected to. This comprises desktop machines as well as mobile devices, and is not restricted by the operating system.

A similar method was introduced in 2016 for Java-Script (Chou 2020). In this publication, Chou proposes overriding the “eval” function in JavaScript by directing the execution to a sandbox environment, and logging what is about to be executed. This method is introduced as a manual debugging method for malware analysts instead of an additional security method for inspection devices.

Currently, no NGFW vendor is known to utilize the content injection method described here. Since no additional research or papers have yet been published on this methodology, its usability in real NGFW environments remains to be seen.

## State-of-the-art of endpoint awareness in NGFWs

Different NGFW vendors have chosen to approach the issue of endpoint awareness in different ways. In this section, we give a high level view of the varying approaches NGFW vendors have chosen to take with endpoint awareness. We have used the public materials from each vendor as the basis, and have not performed any concrete technical review of the products. Table 1 gives a summary of the support from five different NGFW vendors for the different approaches for endpoint awareness presented in this paper.

**Table 1:** Comparison of support of the different endpoint awareness methods for different NGFW vendors

	Palo Alto	Fortinet	Forcepoint	Checkpoint	Cisco
CMDR	Yes	Yes	No	Yes	Yes
Active scanning	No	No	No	No	Yes
Passive scanning	N/A	N/A	Yes	N/A	Yes
Metadata	No	No	Yes	No	No

Many NGFW vendors have chosen to approach endpoint security with a separate endpoint product which is not integrated to their NGFW. *Endpoint Detection and Response*, or *EDR*, is the most popular approach to endpoint protection among NGFW vendors. At least Palo Alto Networks, Fortinet, Checkpoint and Cisco have their own EDR solutions (Palo Alto Networks Inc 2022b; Fortinet Inc 2022b; Check Point Software Technologies Ltd 2022a; Cisco Systems Inc 2022). Based on the public material, it is highly likely that these EDR solutions utilise the same intrusion prevention methods and databases as the NGFW products from the same vendor, but work independently on the endpoint.

CMDBs are also supported by many NGFW vendors. Some vendors, such as Palo Alto and Checkpoint, have native support for specific CMDB products (ServiceNow 2022; Check Point Software Technologies Ltd 2020). Other vendors, such as Fortinet, have added general support for importing CMDB data from an external source (Fortinet Inc 2020).

Active scanning has been implemented directly by some NGFW vendors. This includes WatchGuard Technologies (2020) and Cisco Systems Inc (2020a). Support for Endpoint metadata was found in two network security solutions: the McAfee IPS solution (2020) and Forcepoint (2020).

Since deep packet inspection is at the core of each NGFW, different passive identification methods have been included in many implementations. It is, however, somewhat difficult to find any public information about the different passive endpoint application identification capabilities as the IPS features are often considered proprietary information.

Cisco has had several public references to their TLS fingerprinting capabilities, referencing JA3 by name (Blake Anderson and Cisco Systems Inc 2022). They also mention that any Cisco product which is able to see the different TLS headers from the Client Hello is able to utilise their TLS fingerprint database (Keanini and Cisco Systems Inc 2022). This can be interpreted to indicate that the Cisco Firepower NGFW has this support.

Similar official support could not be found for other vendors that we looked into. However, looking at the public signature databases, we were able to find several references to JA3 fingerprints for Forcepoint NGFW (Forcepoint LLC 2022). We were not able to find any similar references from the Fortinet signature database (Fortinet Inc 2022c) or the Checkpoint signature database (Check Point Software Technologies Ltd 2022b). The Palo Alto signature database is private, and we were unable to verify if they include similar signatures (Palo Alto Networks Inc 2022c).

We were unable to find any public information about an NGFW vendor who would have implemented the content injection method. It is possible that such a feature would be regarded as proprietary information, and thus kept private.

## Open issues

Many methods for passive network traffic fingerprinting use machine learning on large datasets. To apply these methods to an NGFW, the information needs to be generated separately, and the updated information needs to be dynamically imported to the NGFW. To receive up-to-date information, constant iteration on updated datasets needs to be implemented for pattern extraction. Unfortunately, these methods often also require such information on the connection that can only be collected after the whole connection has been processed. This invalidates its usability on inline security. Nonetheless, the traffic patterns are not very labile as shown in Taylor et al. (2018), where information collected six months prior could still be used with 96% accuracy to identify the mobile application.

During this study, it was found that most of the existing academic research on passive endpoint application identification focuses on HTTP and TLS traffic. Since an NGFW is often used for controlling and protecting all network traffic, its scope is not restricted to HTTP or TLS traffic. It was noted, however, that there is a lot of active development ongoing in the field of hash fingerprinting endpoint applications over different protocols. In addition to TLS, we discovered fingerprinting methods for SSH, gQUIC, RDP and SMB. Due to the cutting-edge nature of these methods, no further research has yet been published on their effectiveness, but the preliminary results presented by their developers show promise.

We found that the existing research on passive endpoint application identification focuses almost solely on identifying the client application. As an NGFW is often used for protecting a server installation, it is clear that the current research is heavily lacking in server application identification. The only methods for passively identifying the server application that we encountered were the JA3S and HASSHServer fingerprinting methods which are not well covered in current research.

Regarding active endpoint application identification, we note that using an external software component for providing metadata of the endpoint application to the NGFW is an accurate identification method which does not require the endpoint to compromise its security. Combining this information with traffic fingerprinting methods, like hash fingerprints, and storing the information to be used for identification on other environments seems to be a promising field for further study. The results in Truong (2019), however, pose some issues that need to be taken into account, especially regarding the conflict of different solutions using the same libraries.

We acknowledge the concept of content injection, which is the process of modifying the inspected content in a certain way by the NGFW. This approach provides an interesting method for providing endpoint specific protection, but no research has yet been published on its usability in real life, and no known implementations exist. In addition, depending on implementation method, content injection may produce additional privacy concerns as it involves sending information, which would potentially not have been included in the network traffic otherwise, to the NGFW from the client. It is important to take this into account when considering further implementation of content injection. All in all, further research on the topic is needed.

### Discussion

A big part of the existing research on passively identifying the endpoint application, based on the network traffic, approaches this issue with the assumption that the traffic is observed by someone intending to harm the endpoint. The ability to identify the endpoint application based on network traffic is considered harmful and something to be prevented. This approach ignores the use-case where the interceptor attempts to provide endpoint aware protection. This is, however, a valid concern, and the issue of providing endpoint aware protection in an NGFW should not come with the requirement of reduced security.

Endpoint identification can provide NGFWs in a zero trust network environment additional capabilities for protecting the network according to zero trust principles. More accurate endpoint application identification will support a robust continuous resource assessment process inherent in zero trust, where no assumptions are made regarding a network device based on previous access or approved actions. Situational awareness is an essential part of successful zero trust network implementation (Shore et al. 2021), and improved endpoint identification capabilities for NGFWs will help in successfully implementing zero trust in networks.

Using an external software component on the endpoint for providing metadata on each connection for the NGFW is the most reliable method for accurately identifying the different endpoint applications behind different network connections. It is also secure in the sense that it does not require that the traffic can be passively identified by anyone with visibility to the traffic, and it does not modify the traffic. Collecting traffic patterns like hash fingerprints from environments that utilize such an external component, and using the information for identifying traffic on other environments that do not utilize such a component, provides a constant source for up-to-date endpoint application traffic patterns. However, the accuracy of such fingerprints on legitimate endpoint applications is yet to be extensively studied.

Using machine learning methods for identifying the endpoint application in the case of browsers and mobile applications has proven to be very reliable. Such methods for identifying a client application from TLS traffic have been introduced, for example, in Muehlstein et al. (2017), and mobile application identification has been studied, for example, in Shbair et al. (2016) and Taylor et al. (2016). These methods often rely on features that can only be extracted from the connection after it has been processed. This makes them useless for inline security in an NGFW, but usable for post processing such as network traffic reports. To be useful for inline security, these methods would require that the information is actively generated and collected on a separate environment and dynamically delivered to the NGFWs.

## Conclusion

In this paper, we have performed a thorough study of the existing academic research and state-of-the-art implementations of methods for providing endpoint aware protection in the context of a Next Generation Firewall. We have divided our study into two areas: passive and active endpoint application identification methods. To our best knowledge, this is the first study to examine research and existing implementations of endpoint aware inspection.

When studying the passive endpoint application identification methods, we found that the existing academic research focuses heavily on HTTP and TLS. As an NGFW is used for controlling and protecting all network traffic, there is a significant area for further research for other network protocols. The most significant topic for future research is the QUIC protocol, which is quickly becoming a prominent internet protocol. Regarding passive endpoint application identification, we especially note the promise in hash fingerprinting. Several studies have proven, that the list of supported cipher suites in a TLS Client Hello message is an effective method for identifying the client application, and the JA3 and JA3S hash fingerprints utilize similar methods. However, there is a clear need for further research on the accuracy of JA3 and JA3S fingerprints. In addition to JA3 and JA3S fingerprints, we discovered several other similar hash fingerprinting methods for other protocols that were in a more preliminary stage, but show promise. We also noted that the academic research on identifying the server application is lacking.

When studying active endpoint application identification methods, we note that server application identification, which is lacking on the passive identification side, is well covered with active scanning methods. We acknowledged the value which can be received from integrating a CMDB solution into an NGFW, but note that the information stored on a CMDB may be out of date. We establish that installing an external component on the endpoint, which provides the NGFW with endpoint metadata for each network connection, is a false positive free method for implementing endpoint aware inspection. However, this method requires the ability to install an external component on each endpoint, which is not always feasible. Nonetheless, we note that endpoints with these external components installed provide a unique platform for collecting accurate information on the traffic patterns of endpoint applications. This information can later be used for identifying the endpoint application from traffic patterns, where the metadata cannot be collected. We especially suggest using the endpoint metadata to map hash fingerprints to endpoint applications. Finally, we briefly explore the concept of distributed client protection, which is the method of injecting content to the traffic, affecting how the content gets executed on the endpoint application. We note that this is a promising but insufficiently studied, concept, which requires further study.

In conclusion, we establish that there are many open areas for future research in the endpoint aware inspection methods in the context of a Next Generation Firewall. Based on current research and state-of-the-art implementations, there are no silver bullet solutions: all methods included in this study had their weaknesses, be it in accuracy, invasiveness, or required resources. Based on this study, the best results are achieved when both passive and active methods can be utilized in the protected environment.

## Data Availability

Not applicable.

## Abbreviations

NGFW: Next generation firewall
IPS: Intrusion prevention system
VPN: Virtual private network
FWaaS: Firewall as a service
SD-WAN: Software-defined networking in a wide area network
SASE: Secure access service edge
SVM: Support vector machine
RBF: Radial basis function
CMDB: Configuration management database
CI: Configuration item
EDR: Endpoint detection and response
